# Effect of birth weight and nutritional status on transverse maxillary growth: Implications for maternal and infant health

**DOI:** 10.1371/journal.pone.0228375

**Published:** 2020-01-30

**Authors:** Laura Jackeline Garcia Rincon, Gizelton Pereira Alencar, Marly Augusto Cardoso, Paulo Capel Narvai, Paulo Frazão

**Affiliations:** 1 Department of Politics, Management and Health, School of Public Health, University of São Paulo, São Paulo, Brazil; 2 Department of Epidemiology, School of Public Health, University of São Paulo, São Paulo, Brazil; 3 Department of Nutrition, School of Public Health, University of São Paulo, São Paulo, Brazil; Ohio State University, UNITED STATES

## Abstract

We analyzed the association between birthweight, nutritional status and transverse maxillary growth in 7- to 9-year-old schoolchildren. We undertook a cross-sectional survey nested in a population-based cohort study of 158 schoolchildren. The participants lived in the urban area of a small town within the Western Brazilian Amazon. The outcome was represented by the upper intermolar distance given in millimeters (mm), as an indicator of the degree of maxillary bone growth in its transverse dimension. The exposures were sex, birthweight, the bottle-feeding pattern operationalized by a scale corresponding to the age of introduction of the bottle and Body Mass Index-for-age z-score (BAZ) at 4 to 6 ys. Path analysis was employed to estimate standardized direct, indirect and total effects of exposures on the outcome using structural equations model (SEM) supported by Mplus 7 program. The values of standardized coefficients (SC) showed significant direct positive effects of sex (SC = 0.203; p = 0.006), birth weight (SC = 0.155; p = 0.030) and BAZ (SC = 0.165; p = 0.014) on transverse maxillary growth. The indirect effects (SC = 0.057; p = 0.012) and the total effect (SC = 0.261; p<0.001) of sex on the outcome were statistically significant. The indirect effects of birth weight on the outcome were not significant (SC = 0.018; p = 0.488), however, the total effect was significant (SC = 0.174; p = 0.011). In conclusion, sex, birthweight, bottle beginning age and BAZ showed association with the transverse growth of the maxillary bone. In addition to contributing to an adequate birth weight of the child, policies and programs that favor prenatal care and conditions to guarantee a full-term birth can positively affect transverse growth of the maxilla. From a Public Health Surveillance point of view, children with reduced birthweight, inadequate breastfeeding pattern and nutritional deficit for age may be more likely to develop atrophy of the jaws which, depending on the severity, may result in malocclusion with an important impact on quality of life.

## Introduction

Malocclusion is a multifactorial alteration of the normal growth and development with effects on muscles and facial bones during childhood and adolescence; it is a collection of situations, any of which can aggravate due to genetic predisposition or external factors. It can affect the oral functions, such as mastication, swallowing and speech; it implicates greater susceptibility to trauma and periodontal disease; and it can also have an important psychosocial repercussion due to impaired dentofacial esthetics.

Although much epidemiological information is the result of the analysis of cross-sectional studies, including the possible bias inherent in such research, it is recognized that the prevalence and severity of malocclusions have increased in the last 200 years, especially dental crowding, which is the most prevalent type of malocclusion [1, 2]. Malocclusion such as posterior crossbite, dental crowding and asymmetries may occur because of transverse changes of the maxillary bones [3] and the upper intermolar distance may be used as an indicator of transverse growth of the maxilla [4].

The maxilla is a complex anatomical structure whose growth process is given by multiple factors, such as sutural growth until 7 years old. After this age it gets slower and is more characterized by areas of apposition and resorption, simultaneously accompanied by displacement forward and downward. It is accompanied by a calcification process, tooth eruption and the intervention of muscular forces that harmoniously promote proper development. The eruption of the teeth and the consequent growth of the alveolar process will increase the vertical dimension of the maxilla. With the eruption of the first permanent molars begins the mixed dentition, which extends from 6 to 12 years, and it is a period of particular importance in the etiology of malocclusion since during these years a series of interconnected processes lead to changes of temporary by permanent dentition; and definitive occlusion is established. At 6 years old, the upper first permanent molars erupt behind the second temporary molar. During the time of temporary dentition the width of the dental arch increases slightly. The main increase of the arch occurs by growth in a posterior direction as the teeth erupt, this expansion is detected in the same way in permanent dentition. The expansion of the upper dental arch width is greater than in the lower one [5].

McNamara reported normal maxillary width of 35mm in mixed dentition measured from the lingual surfaces. Values from 36 to 39 mm could accommodate a dentition of average size without crowding or spacing, whereas maxillary arches less than 31 mm in width might be crowded and thus in need of orthopedic or surgically assisted expansion [6].

Potential factors associated with changes in maxillary growth include genetic traits (which may also determine the presence of craniofacial syndromes), mouth breathing, decreased oral functions, premature loss of temporary teeth, infantile facial paralysis, delayed onset of complementary feeding, muscular hyper or hypotonicity (in the orbicular muscles of the mouth), non-nutritive sucking habits, atypical phonation and swallowing, and abnormal tongue position or other environmental factors, thus demonstrating an interaction *nature and nurture* [7, 8]. These factors are in permanent interaction and together promote the conditions for the growth of craniofacial complex and thus determine the facial growth pattern.

Previous studies conducted on this subject are limited, therefore epidemiological information is scarce and mostly generated from cross-sectional designs preventing a view on the role of certain characteristics of the child's life course, a perspective that has become important with the emergence of theories about the early life influence in the health status in later years and in the rest of life. It has been hypothesized that aspects of the perinatal period and childhood could promote a "biological programming" associated with development of chronic diseases years later. David Barker, one of the pioneers in this subject, proposed that the first stages of early life were a critical period that permanently programs body metabolism and growth, and thus determines pathologies in adulthood, in addition to being related to future socioeconomic conditions. Several relationships between circumstances of early life and consequences at different times throughout life have been studied [9, 10].

The aim of the study was to investigate the factors associated with transverse growth of maxillary bone, expressed by the upper intermolar distance, from a point of view related to the course of life.

## Materials and methods

### Study area and population

The study was approved by the Institutional Review Board of the School of Public Health, University of Sao Paulo, Brazil (297/2009). Written informed consent was obtained from the parents or guardians of children who participated in the study before enrollment. Data were analyzed anonymously.

It links to a larger research project on health and nutrition conditions at Acre state developed by Sao Paulo University and Acre Federal University, whose field activities began with population-based cross-sectional surveys in 2003 in the urban area of a small town within the Western Brazilian Amazon [11]. The characteristics of the place and cross-sectional studies were described previously [12, 13].

The reference population described elsewhere [14] comprised all children 7–9 yr of age, enrolled in urban schools in 2010 and who had participated in the 2007 census household survey. The children were from families that took their livelihood from agriculture and extractivism. The characteristics of the study territory were similar to those territories with illiteracy rate between 20 and 30%, medium human development (0.555–0.699) and infant mortality rate between 30 to 35 deaths per 1,000 live births.

Schoolchildren of both sexes, with no extensive occlusal caries in upper permanent molars; no history of orthodontic or major surgical treatment; no congenitally missing teeth; no signs or symptoms of temporomandibular joint disorder, no mouth breathing, no diseases such as systemic and/or neurological ones and those whose responsible person accepted the dental examination, were eligible for the study.

### Variables of the study

The dependent variable was represented by the upper intermolar distance given in millimeters, measured between the center point of the occlusal surface of the right and left first permanent molars (mm), assuming that it can operate as an indicator of the degree of maxillary bone growth in its transverse dimension [4, 5]. In 2010, dental examinations were performed by a single dentist under natural lighting conditions and using a drypoint caliper and a Mimatoyo digimatic caliper with accuracy of +/- 0.02mm. Before the examinations, the dentist was trained by a specialist from Public Health School at University of São Paulo. The examiner underwent to theoretical sessions in which one expert on oral health surveys conducted the discussion about the premises related to the adopted criteria using intraoral photographs of specific situations and cases, and oral examination sessions at a non-sampled school in São Paulo city. The consistency of the occlusal examinations carried out in the research field was measured using standardized study models made of gypsum because they are able to copy as faithfully as possible the dental structures and the accuracy of the intermolar distance studied here is a critical factor of this research. Children with all primary canine teeth and all first permanent molars in hygienic conditions were considered eligible for impression of both upper and lower dental arches with irreversible hydrocolloid (alginate) and the design of the study models. These models were used to obtain indirect measures related to the outcome. The values obtained through the indirect measurements were compared with the values of the direct measurements using the intraclass correlation coefficient (ICC). Thirty-seven models were obtained for controlling intra-examiner consistency. The values of the direct and indirect linear measurements for the intermolar distance were compared and the ICC value was 0.713 (CI = 0.509–0.841, p<0.001).

**Fig 1:**
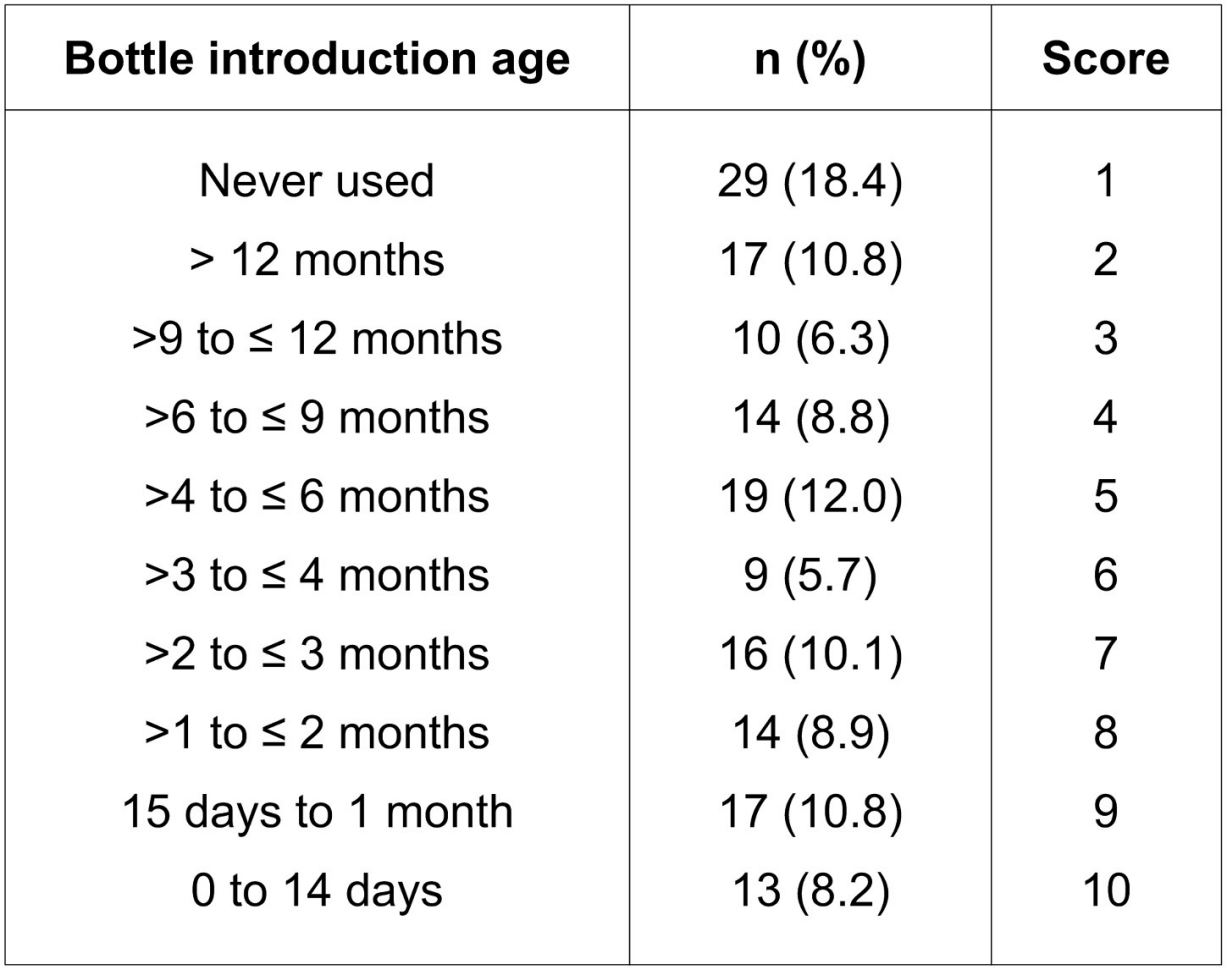
Bottle-feeding pattern scale elaborated from the bottle introduction age for children from 7 to 9 years old in the Western Brazilian Amazon.

The independent variables were related to the characteristics of the child. Sex, birth weight and the bottle-feeding pattern operationalized by a scale corresponding to the age of introduction of the bottle, were obtained in a population-based cross-sectional study involving children aged 0.1–5.5 years at 2003 [11]. The bottle-feeding scale was build based on the distribution close to deciles of age of bottle introduction measured in days (Fig 1). In 2007, height and weight were measured by trained research assistants following standardized procedures and using calibrated equipment in a population-based cross-sectional study when the study sample had from 4 to 6 years-old [15]. In brief, children were measured to the nearest 0.1 cm when standing barefoot in a stadiometer (model 208; SECA, Hamburg, Germany). Weight readings were taken on an electronic scale (model HS-302; Tanita, Tokyo, Japan) and recorded to the nearest 100 g. Each measurement was repeated once, and the mean value was calculated. Body mass index (BMI) was computed as weight in kg divided by height in square meters. Z-scores for BMI were calculated in accordance with the WHO child growth standards for children aged 0 to 5 years [16] and the WHO Growth Reference Data for children >5 years of age [17].

### Statistical analysis

The descriptive analysis of the data was performed with the program Stata/SE 13.1. It was tested adherence to the normal curve of the dependent variable through the Kolmogorov-Smirnov test. The t-test was performed to compare the mean values of the intermolar distance between sexes, as well as differences in birth weight and BMI-for-age z-score between sexes.

Statistical analyzes of the data were carried out using the path analysis approach in the framework of a structural equations model (SEM) with aid of the Mplus 7 program [18]. The Robust Maximum Likelihood estimation (MLR) was applied to analyze the structural relationships, corresponding to the associations between variables expressed by standardized coefficients (SC). The p-values of the associations were set at 0.05.

**Fig 2:**
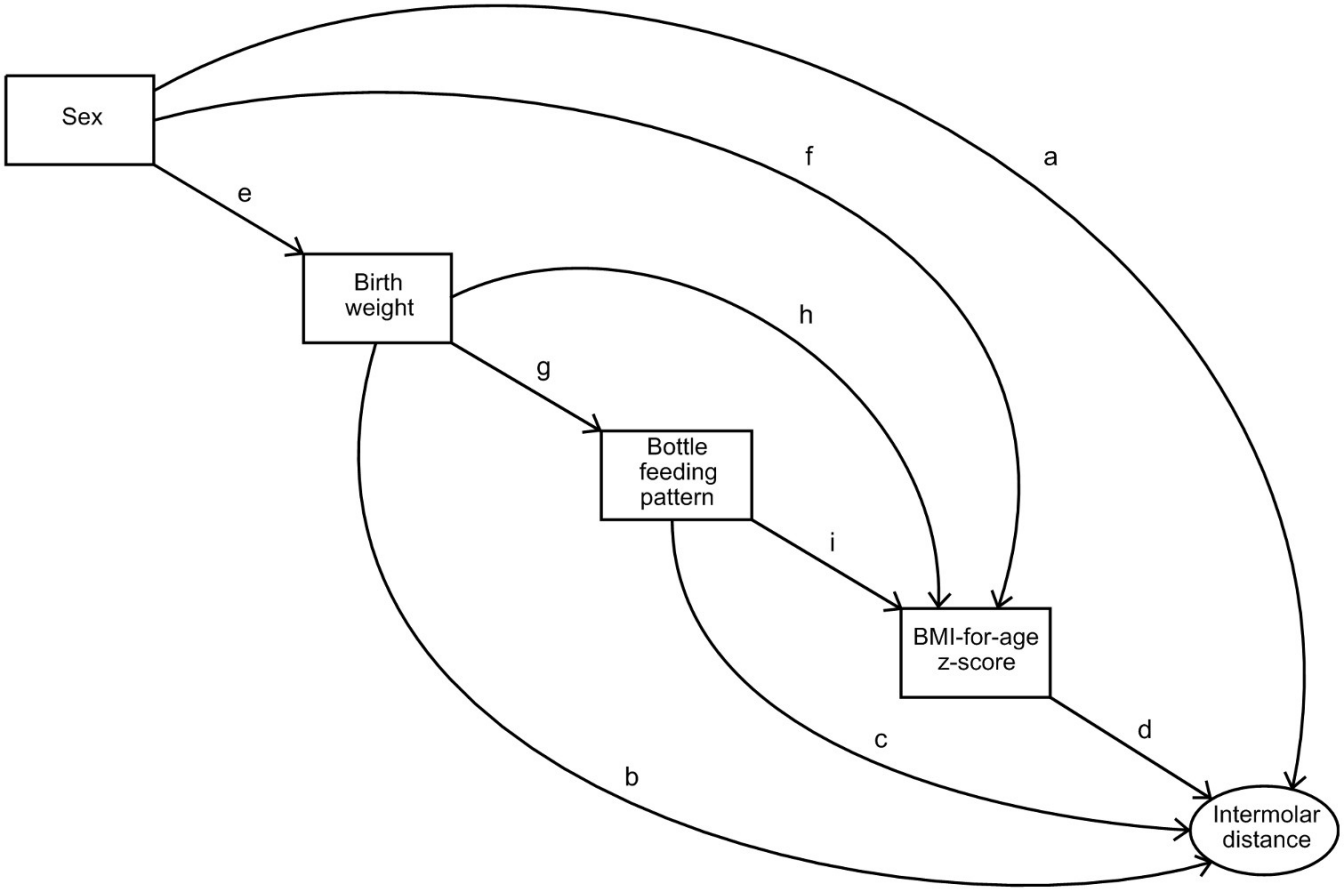
Theoretical model of the different paths that were tested between the independent variables and the association with the upper intermolar distance. References: **a**. [20, 21, 34, 35, 36] **b**. [22–24] **c**. [25, 37, 38] **d**. [39, 40] **e**. [40, 41] **f**. [15] **g**. [29] **h**. [15, 29] **i**. [31].

This modelling allows to verify a theoretical model, in which the hypothesized relationships among variables are tested in a linear equation system. A variable can be defined as an outcome variable in one relation and as an explanatory variable in another relation. Fig 2 shows the theoretical model that was tested considering some relationships described or suggested in the literature and even some hypotheses not described.

The study of these multiple relations allows testing several hypotheses where an association of these variables with the growth of the maxillary bone, would be present. Firstly, as in previous studies, boys are expected to have a higher birth weight than girls [19]. Children who had a higher birth weight could have a better nutritional status during childhood compared to children with reduced birth weight. Studies that have found sexual dimorphism for some vertical craniofacial dimensions allow us to hypothesize that this sexual dimorphism may also manifest in transverse maxillary growth [20, 21]. Children with low birth weight have smaller dental crowns and poor-quality enamel [22–24]. This may result in the hypothesis that children with lower birth weight have the potential to present smaller maxillary bones.

The hypothesis supporting an association between bottle beginning age and transverse maxillary growth was based on studies that have produced evidence that bottle-feeding is detrimental to the development and function of oral maxillofacial structures [25–28]. In addition to impairing the duration of exclusive breastfeeding, it contributes to early weaning and induces the child to form non-nutritive sucking habits through nozzles and pacifiers (not being the unique cause). These end up favoring changes in the morphology of facial bones, also altering the relationships between jaws and teeth, giving rise to occlusion deviations (such as anterior open bite and atresic palate). As the ideal diet for a child under one year old is breastfeeding [29], children who are exposed to bottle at very early ages, can develop nutritional deficiencies that impact on general bone growth. These deficiencies can imply a reduced space in the jaws for dental eruption. In view of this situation, we investigated a possible association between bottle introduction age and upper intermolar distance as an estimator of transverse maxillary growth.

The adjustment of the structural equations model was performed using the goodness of fit indicators: Comparative Fit index (CFI) with reference value higher than 0.95; Tucker Lewis Index (TLI) with values above 0.95; and the root mean square errors of approximation (RMSEA) expecting values less than 0.05.

As no previous information is available, the study was considered exploratory. No a priori hypothesis was admitted about presumed associations and no study power calculation was performed hoping that subsequent research can be designed with greater precision knowledge.

## Results

### Sample characteristics

The study sample selected from the reference population consisted of 158 children between 7 and 9 years of age, of whom 73 were male (53.8%) and 85 were female (46.2%). Thirty-seven were no eligible and twenty-three had missing data on specific variables. The age distribution in the sample was given by 48 children aged 7 years (30.4%), 62 children aged 8 years (39.2%) and 48 children aged 9 years (30.4%). A proportion of 8.9% (n = 14) of children with low birth weight (< 2,500 g) was found. Of these, 10 (71.4%) were female.

Table 1 shows descriptive statistics of the study sample. Significant correlations were observed among exposure variables and the outcome. The mean birth weight of the boys (3,490.75 g) was higher than that of the girls (3,185.72 g) and the difference was statistically significant (p = 0.003). The t-test showed that for boys the average birth weight was 305 g higher than for girls (p = 0.003). The mean BMI-for-age z-score among boys was 0.12 and among girls at 4 to 6 ys was -0.18 with a near significant statistical difference (p = 0.057).

**Table 1:** Descriptive statistics of study variables in children aged 7 to 9 years. Western Brazilian Amazon, 2010.

Variable	Mean (SD)	Median	Min-Max	r*	p-value
**Intermolardistance (mm)**	45.38 (2.97)	45.56	31.71; 53.43	-	-
**Sex**	-	-	-	0.27	0.001
**Birthweight (g)**	3,326.65 (642.03)	3,335.00	1,200.00; 4,730.00	0.23	0.004
**Bottlefeedingpatternscale**	5.09 (3.06)	5.00	1.00; 10.00	-0.16	0.051
**BMI-for-age z-score**	-0.04 (0,99)	-0.14	-2.43; 3.09	0.24	0.003

* Pearsoncorrelation coefficient between each independent variable and the upper intermolar distance

The upper intermolar distance showed adherence to the normal distribution (p = 0.625). When comparing the mean values of the upper intermolar distance between the two sexes, a statistical difference was found (p = 0.001); with the mean in boys being 46.25 mm and in girls being 44.63 mm. Comparing the differences between the mean values of the upper intermolar distance between the children's ages (7, 8 and 9 years), there were non-significant statistical differences.

**Fig 3:**
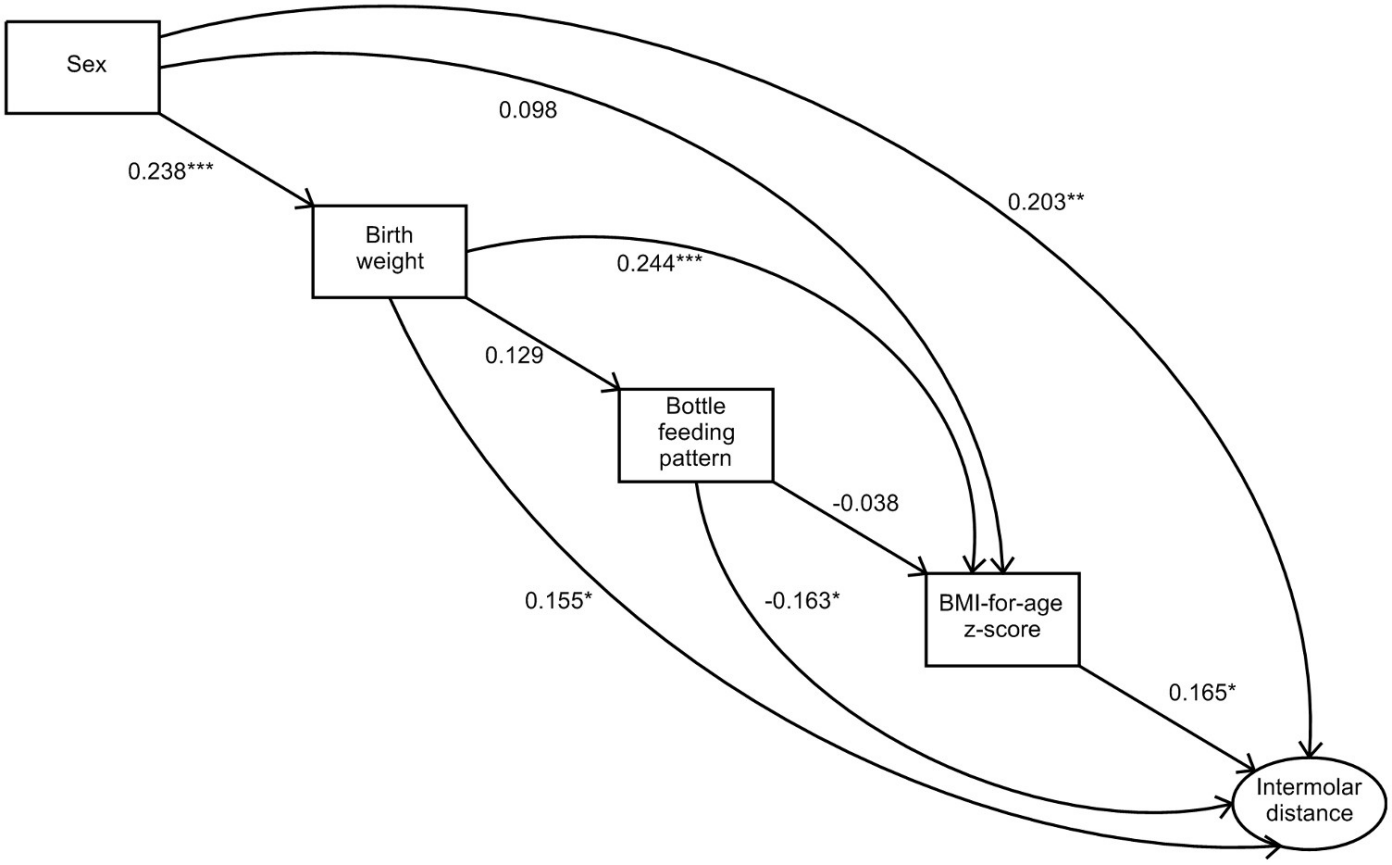
Structural equations model for the upper intermolar distance of children between 7 and 9 years old. Acrelandia, Western Brazilian Amazon. 2010. Level of significance * <0.05 ** <0.01 *** <0.005.

Several relations between the variables, both direct and indirect, were observed in the SEM (Fig 3). The model fit was considered satisfactory: CFI (0.995); TLI (0.952); RMSEA (0.037).

The four independent variables considered in the study (sex, birth weight, bottle-feeding pattern scale and BMI-for-age z-score) had a direct and significant association with upper intermolar distance. The overall effect of sex on the outcome was significant (SC = 0.261, p<0.001). The direct positive effect of sex on the intermolar distance reflects that the boys presented higher values of this measure indicating, therefore, a greater transverse growth of the maxillary bone in this group (SC = 0.203; p = 0.006). The t-test showed that for boys the average intermolar distance was 1.627 mm higher than for girls (p = 0.001).

The positive direct effect of birth weight on the outcome indicates that the higher the birth weight of the child, the higher the intermolar distance (SC = 0.155, p = 0.030). Likewise, the higher the BMI-for-age z-score, the higher the intermolar distance (SC = 0.165, p = 0.014).

The bottle-feeding pattern scale showed a negative direct effect on the outcome. Given that this variable assigns a value from 1 to 10 (where 1 is for those who never used a bottle and 10 for who started to use a bottle between 0 and 14 days of age) as seen in Fig 1; i.e. the earlier the bottle was introduced, the lower the value observed for the upper intermolar distance (SC = -0.163, p = 0.013).

The indirect effects produced by the variables on the outcome can be seen through several paths among the independent variables. Starting with sex, which is the most distal variable with respect to intermolar distance, we observed the direct effect and five ways that contribute to the indirect effects of this variable on the object of study. As shown previously, the direct effect was significant (SC = 0.203, p = 0.006). The set of the five indirect effects was also significant (SC = 0.057, p = 0.012). However, when analyzing each indirect effect separately, it is observed that only one of them of them was significant (Table 2).

**Table 2:** Indirect effects of the sex variable on the upper intermolar distance in children from 7 to 9 years of age. Western Brazilian Amazon, 2010.

Analyzed path	Coefficient	p-value
Sex TO BMI-for-age z-score TO upper intermolar distance	0.016	0.270
Sex TO birth weight TO upper intermolar distance	**0.037**	**0.048**
Sex TO birth weight TO BMI-for-age z-score TO upper intermolar distance	0.010	0.090
Sex TO birth weight TO bottle feeding pattern TO upper intermolar distance	-0.005	0.213
Sex TO birth weight TO bottle feeding pattern TO BMI-for-age z-score TO upper intermolar distance	0.000	0.633

Regarding the effects of birth weight, besides the direct effect, two paths were found through which indirect effects were observed. As shown previously, the direct effect of birth weight on the upper intermolar distance was statistically significant (SC = 0.155, p = 0.030). The indirect effects together (SC = 0.018, p 0.508) or each path separately did not show statistical significance (Table 3), but the total effect of birth weight on the outcome was statistically significant (SC = 0.174, p = 0.011).

**Table 3:** Indirect effects of the variable birth weight on the upper intermolar distance in children from 7 to 9 years of age. Western Brazilian Amazon, 2010.

Analyzedpath	Coefficient	p-value
Birth weight TO BMI-for-age z-score TO upper intermolar distance	**0.040**	**0.036**
Birth weight TObottle feeding pattern TO upper intermolar distance	-0.021	0.159
Birth weight TO bottle feeding pattern TO BMI-for-age z-score TO upper intermolar distance	-0.001	0.625

Bottle feeding pattern showed an indirect effect way with no statistical significance (SC = -0.006; p = 0.619). The total effect that summarizes direct and indirect effects was significant (SC = -0.169; p = 0.010).

## Discussion

Sex, birth weight, bottle-feeding pattern and BAZ were associated with upper intermolar distance. The effect of each variable on the outcome was expressive. In addition, indirect effects of these variables on the upper intermolar distance were identified.

As strengths of the work, the characteristic of being a cross-sectional study nested in a cohort and the utilizing of structural equation models could be quoted, since this combination allows to determine the temporal sequence and to study several regression models simultaneously for establishing direct and indirect relationships among the considered variables.

Although the study has yielded new insights into the growth of the maxilla associated with nutritional and health determinants in early childhood, and may be a useful tool for further research to be developed in populations with other characteristics, some limitations should be highlighted. Socioeconomic variables, skeletal pattern, altered jaws and teeth sizes, type of respiration and swallowing, complementary feeding and non-nutritive sucking habits were not investigated at first stages of children’s development; and it would be quite important to study these aspects. Excepting non-nutritive sucking habits, there are scarce information about their impact on the outcome. Impaired oral functions could be etiological factors of the malocclusion as pressures and forces exerted during these functions could influence the growth of the jaws and tooth eruption [7]. When atypical swallowing movements are developed, other movements arise as a form of compensation. Therefore, the tongue and other muscles experience high pressures and out of normal balance forces. These restricted positions contribute to high narrow palate, posterior cross bite and malocclusion [30]. Nasal breathing is crucial for the proper development of the oral cavity, so the obstruction of the upper respiratory airway could modify the orofacial growth, resulting in typical features such as long face and narrow maxilla; causing a transverse maxillary skeletal deficit and crossbite [31]. Therefore schoolchildren with sings of mouth breathing were not included in this study. In spite of these points, the present study made use of an interdisciplinary approach that involved health professionals from different areas, and whose results have important implications for maternal and child health.

The effect of sex on upper intermolar distance had a significant overall effect with higher values for boys. These results are consistent with those obtained by Aznar et al. from a cross-sectional study in a sample of 1,297 children aged 3 to 6 years in Spain. Their results pointed to canine and intermolar distances—both in the maxilla and mandible—significantly higher in boys. However, the authors did not raised hypotheses to explain it [32]. Previous studies have shown that there are occlusal differences between girls and boys due to sexual dimorphism in craniofacial parameters [20, 21]. Examining 1,094 cephalometrics of individuals with class III malocclusion, researchers identified that between 7 and 9 years of age, girls had significantly smaller linear dimensions of maxilla, mandible and facial height compared to boys' measurements [20].

In addition to the direct effect of sex on the upper intermolar distance, five indirect effects were found in the structural model, which together showed statistical significance. These findings demonstrate that maxillary growth depends on multiple factors, in addition to the genetic influence that is always present. The results regarding the way in which birth weight influences transverse maxillary growth, indicate that the manifestations on the outcome depend on a combined action due to the duration of breastfeeding and physical characteristic of the child measured by BMI-for-age z-score.

Monitoring birth weight is important to avoid risks related to neonatal and infant morbidity and mortality. However, the relationship of birth weight and transverse maxillary growth had not yet been explored. Regarding the effects of birth weight on the outcome, it was observed that the birth weight showed significance by itself and also in the presence of other variables, suggesting that the birth weight influences the transverse growth of the maxilla. Although the indirect effects of birth weight on the upper intermolar distance were not significant, the total effect was.

As expected, boys presented a higher birth weight than girls. Infants with a higher birth weight could have higher values of BMI during childhood compared to those with reduced birth weight. Children with low birth weight have smaller dental crowns and poor-quality enamel [22–24]. This could mean that children with lower birth weight have the potential to present smaller maxillary bones as shown in current study for upper intermolar distance. An open point for future studies would be to investigate the relationships between birth length and the upper intermolar distance.

Association between the infant feeding pattern and the BMI z-score has been previously studied which varies as the duration and exclusivity of breastfeeding changes and the introduction of other types of foods occur. The association changes in magnitude as the feeding pattern changes as well [33]. In this study, such association was not significant likely because some factors were not investigated here, such as introduction of semisolid foods that could be more explored by future studies in other circumstances.

There is evidence of the detrimental effect of bottle feeding on the development and function of oral maxillofacial structures [25–28]. It can prejudice the duration of exclusive breastfeeding, contributes to early weaning and induces the child to form non-nutritive sucking habits. These end up favoring variations in the morphology of facial bones, or even palate bone dimensions, also altering the occlusion. As the ideal diet for a child under one year old is breastfeeding [29], children who are exposed to bottle at very early ages, can develop nutritional deficiencies that impact on general bone growth. These deficiencies can imply a reduced space in the jaws for dental eruption, consequence of a smaller maxillary bone. The concrete link between maxillary growth and upper intermolar distance had not been examined before. In according to the obtained results, the higher score in the bottle-feeding pattern scale, the lower the values for the upper intermolar distance. This means that the earlier the introduction of bottle feeding, the smaller transverse maxillary dimensions. The lack of breastfeeding could have the potential to reduce transverse maxillary growth, as it is the most natural and correct way to get a proper growth and development of the craniofacial complex and stomatognathic system.

In conclusion, girls and boys with reduced birth weight, very early bottle beginning age and diminished BAZ are more likely to have decreased transverse maxillary growth. The results suggest that policies and programs of child health promotion can have a significant effect on transverse maxillary growth contributing to the control of malocclusions such as posterior crossbite, dental crowding and facial asymmetries.

## Supporting information

S1 Data set (XLSX)
